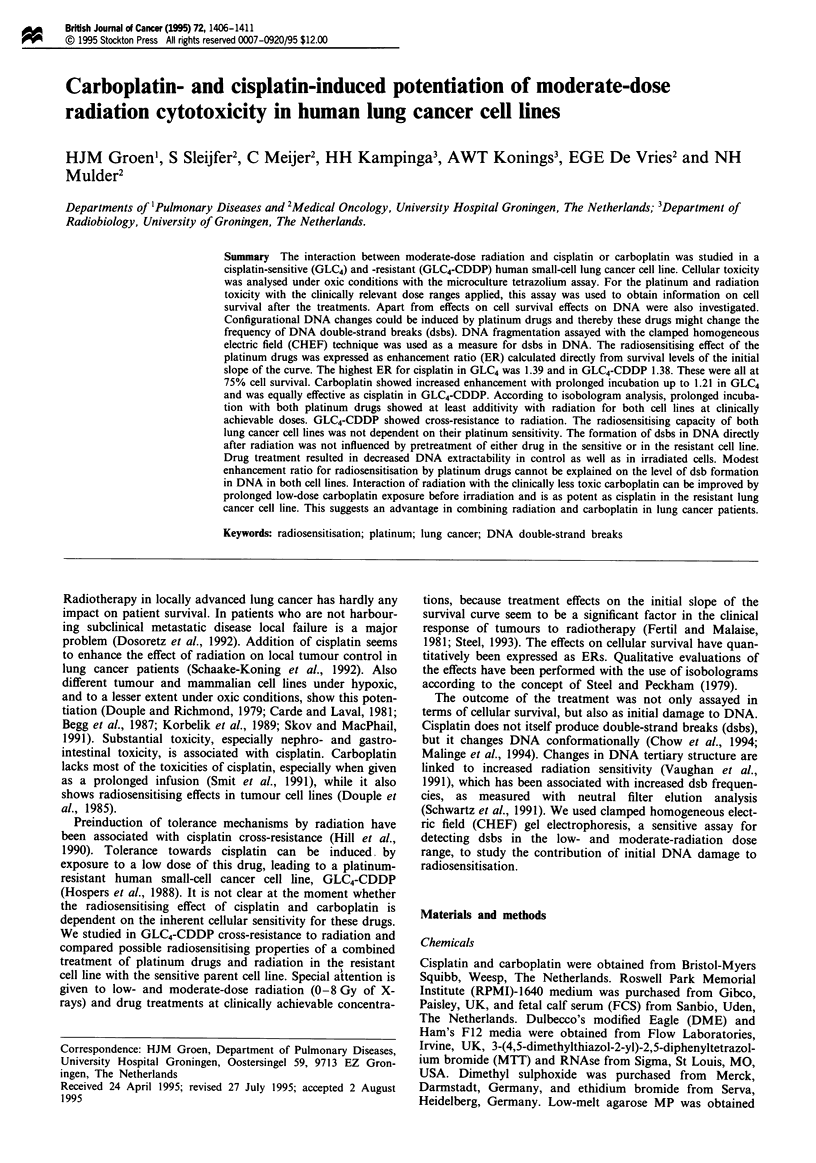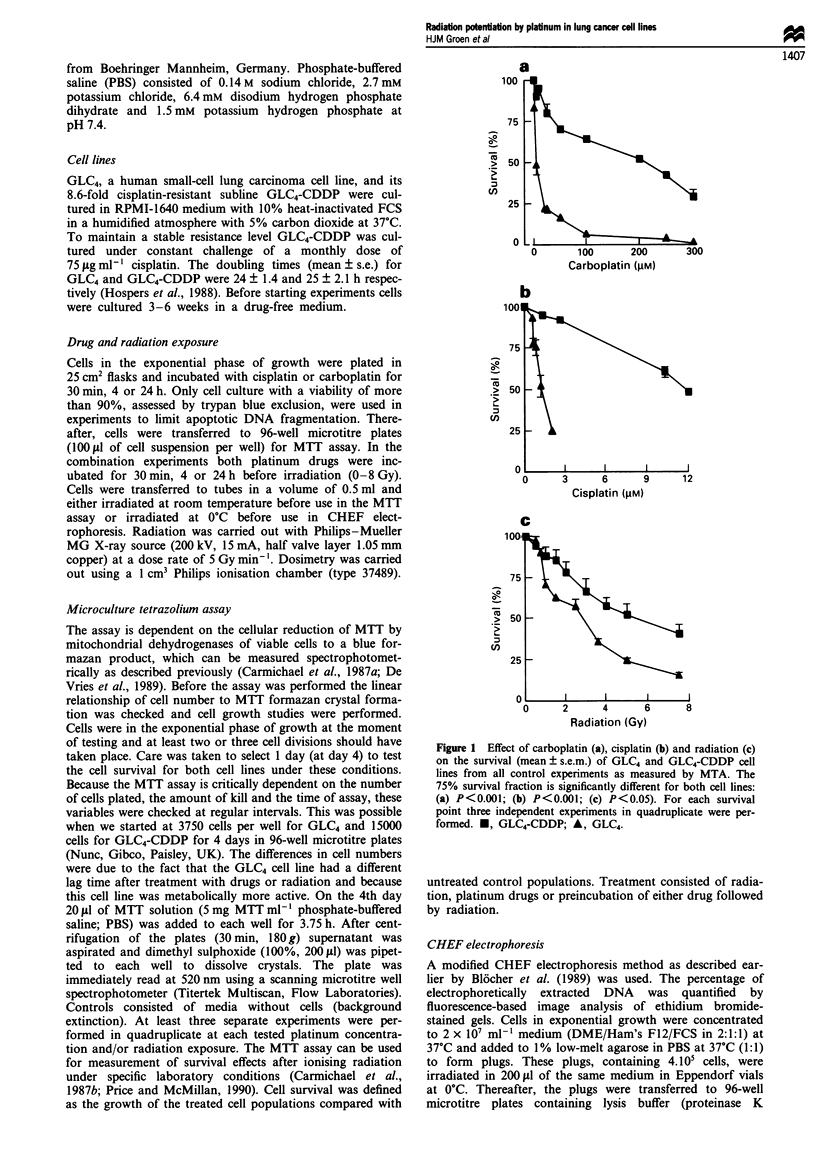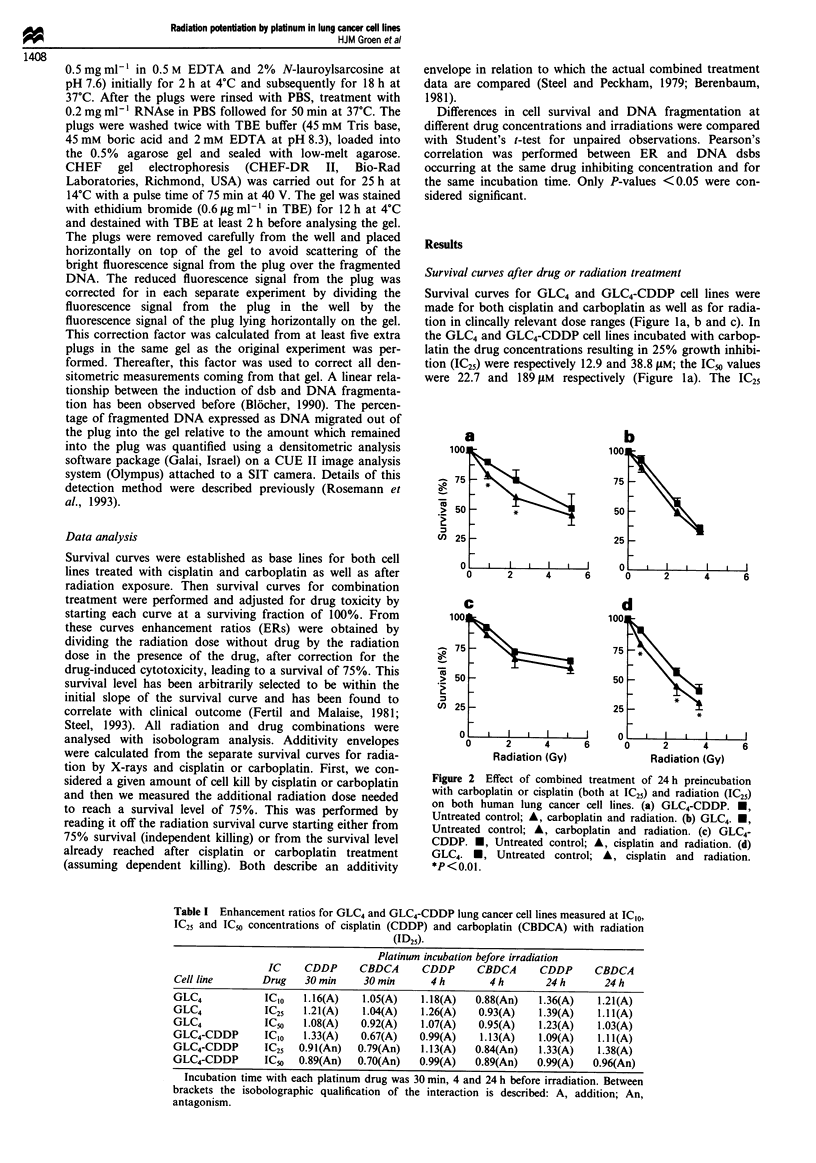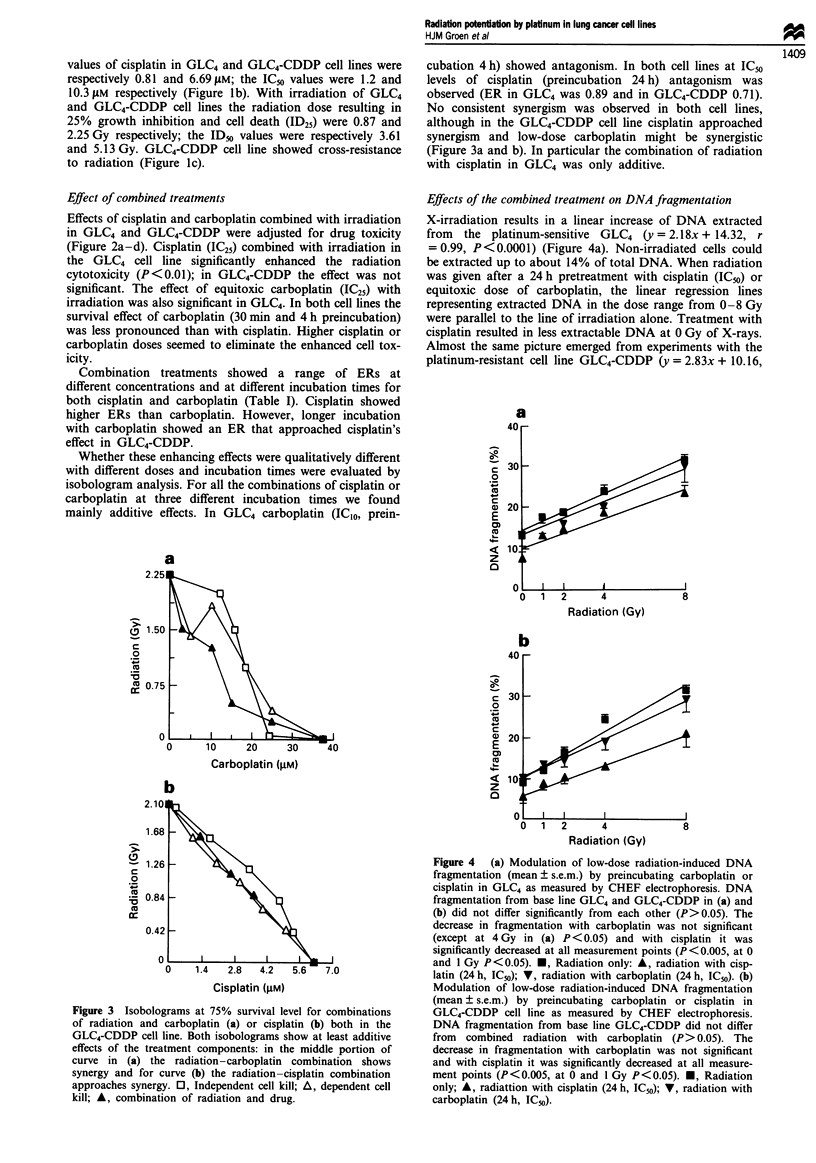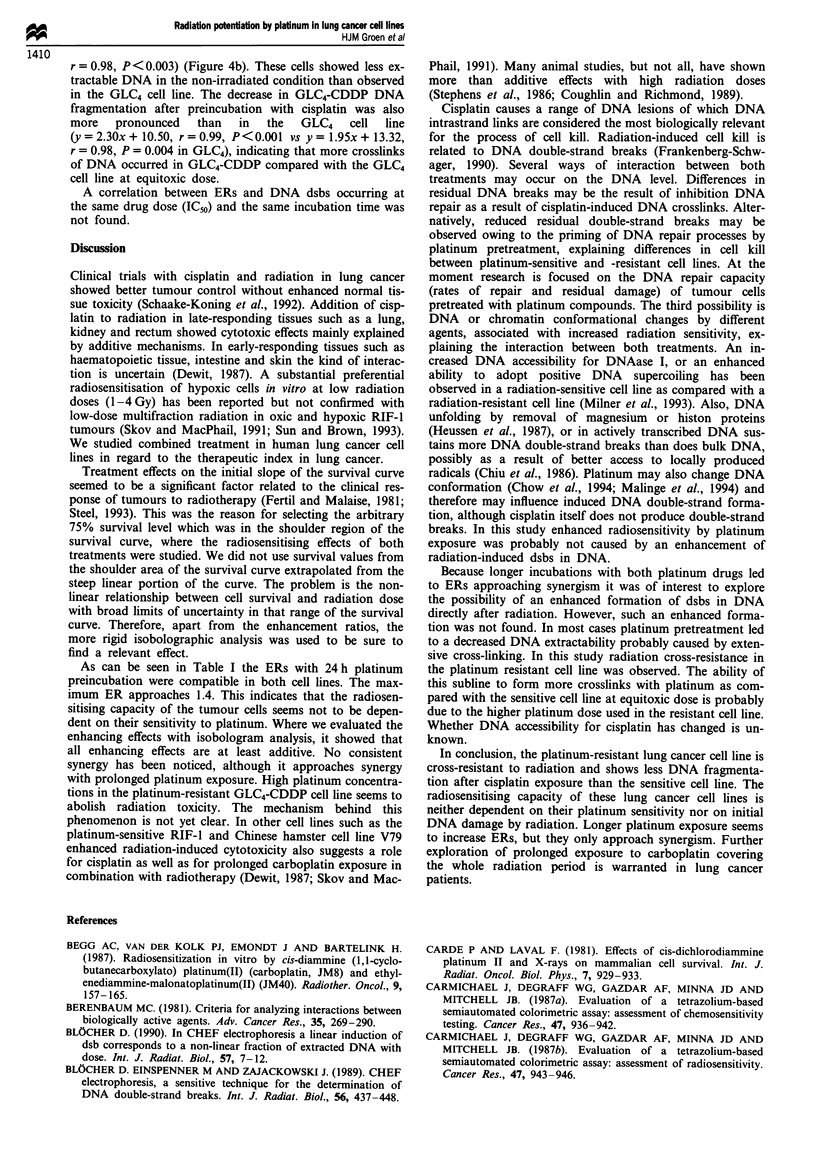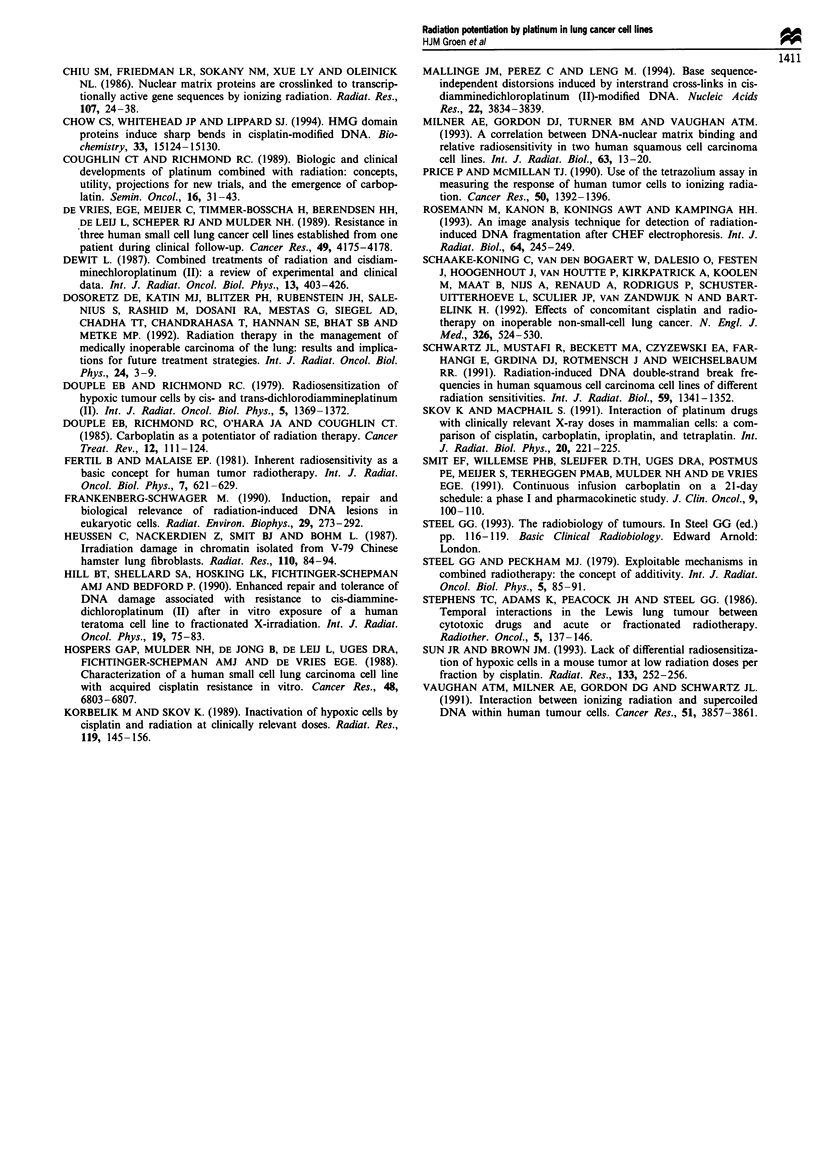# Carboplatin- and cisplatin-induced potentiation of moderate-dose radiation cytotoxicity in human lung cancer cell lines.

**DOI:** 10.1038/bjc.1995.522

**Published:** 1995-12

**Authors:** H. J. Groen, S. Sleijfer, C. Meijer, H. H. Kampinga, A. W. Konings, E. G. De Vries, N. H. Mulder

**Affiliations:** Department of Pulmonary Diseases, University Hospital Groningen, The Netherlands.

## Abstract

The interaction between moderate-dose radiation and cisplatin or carboplatin was studied in a cisplatin-sensitive (GLC4) and -resistant (GLC4-CDDP) human small-cell lung cancer cell line. Cellular toxicity was analysed under oxic conditions with the microculture tetrazolium assay. For the platinum and radiation toxicity with the clinically relevant dose ranges applied, this assay was used to obtain information on cell survival after the treatments. Apart from effects on cell survival effects on DNA were also investigated. Configurational DNA changes could be induced by platinum drugs and thereby these drugs might change the frequency of DNA double-strand breaks (dsbs). DNA fragmentation assayed with the clamped homogeneous electric field (CHEF) technique was used as a measure for dsbs in DNA. The radiosensitising effect of the platinum drugs was expressed as enhancement ratio (ER) calculated directly from survival levels of the initial slope of the curve. The highest ER for cisplatin in GLC4 was 1.39 and in GLC4-CDDP 1.38. These were all at 75% cell survival. Carboplatin showed increased enhancement with prolonged incubation up to 1.21 in GLC4 and was equally effective as cisplatin in GLC4-CDDP. According to isobologram analysis, prolonged incubation with both platinum drugs showed at least additivity with radiation for both cell lines at clinically achievable doses. GLC4-CDDP showed cross-resistance to radiation. The radiosensitising capacity of both lung cancer cell lines was not dependent on their platinum sensitivity. The formation of dsbs in DNA directly after radiation was not influenced by pretreatment of either drug in the sensitive or in the resistant cell line. Drug treatment resulted in decreased DNA extractability in control as well as in irradiated cells. Modest enhancement ratio for radiosensitisation by platinum drugs cannot be explained on the level of dsb formation in DNA in both cell lines. Interaction of radiation with the clinically less toxic carboplatin can be improved by prolonged low-dose carboplatin exposure before irradiation and is as potent as cisplatin in the resistant lung cancer cell line. This suggests an advantage in combining radiation and carboplatin in lung cancer patients.


British Journal of Cancer (1995) 72, 1406-1411
© 1995 Stockton Press All rights reserved 0007-0920/95 $12.00

### Carboplatin- and cisplatin-induced potentiation of moderate-dose radiation cytotoxicity in human lung cancer cell lines

HJM Groen[1], S Sleijfer[2], C Meijer[2], HH Kampinga[3], AWT Konings[3], EGE De Vries[2] and NH Mulder[2]

*Departments of [1]Pulmonary Diseases and [2]Medical Oncology, University Hospital Groningen, The Netherlands; [3]Department of Radiobiology, University of Groningen, The Netherlands.*

**Summary** The interaction between moderate-dose radiation and cisplatin or carboplatin was studied in a cisplatin-sensitive ($GLC_4$) and -resistant ($GLC_4$-CDDP) human small-cell lung cancer cell line. Cellular toxicity was analysed under oxic conditions with the microculture tetrazolium assay. For the platinum and radiation toxicity with the clinically relevant dose ranges applied, this assay was used to obtain information on cell survival after the treatments. Apart from effects on cell survival effects on DNA were also investigated. Configurational DNA changes could be induced by platinum drugs and thereby these drugs might change the frequency of DNA double-strand breaks (dsbs). DNA fragmentation assayed with the clamped homogeneous electric field (CHEF) technique was used as a measure for dsbs in DNA. The radiosensitising effect of the platinum drugs was expressed as enhancement ratio (ER) calculated directly from survival levels of the initial slope of the curve. The highest ER for cisplatin in $GLC_4$ was 1.39 and in $GLC_4$-CDDP 1.38. These were all at 75% cell survival. Carboplatin showed increased enhancement with prolonged incubation up to 1.21 in $GLC_4$ and was equally effective as cisplatin in $GLC_4$-CDDP. According to isobologram analysis, prolonged incubation with both platinum drugs showed at least additivity with radiation for both cell lines at clinically achievable doses. $GLC_4$-CDDP showed cross-resistance to radiation. The radiosensitising capacity of both lung cancer cell lines was not dependent on their platinum sensitivity. The formation of dsbs in DNA directly after radiation was not influenced by pretreatment of either drug in the sensitive or in the resistant cell line. Drug treatment resulted in decreased DNA extractability in control as well as in irradiated cells. Modest enhancement ratio for radiosensitisation by platinum drugs cannot be explained on the level of dsb formation in DNA in both cell lines. Interaction of radiation with the clinically less toxic carboplatin can be improved by prolonged low-dose carboplatin exposure before irradiation and is as potent as cisplatin in the resistant lung cancer cell line. This suggests an advantage in combining radiation and carboplatin in lung cancer patients.

**Keywords:** radiosensitisation; platinum; lung cancer; DNA double-strand breaks

Radiotherapy in locally advanced lung cancer has hardly any impact on patient survival. In patients who are not harbouring subclinical metastatic disease local failure is a major problem (Dosoretz *et al.*, 1992). Addition of cisplatin seems to enhance the effect of radiation on local tumour control in lung cancer patients (Schaake-Koning *et al.*, 1992). Also different tumour and mammalian cell lines under hypoxic, and to a lesser extent under oxic conditions, show this potentiation (Douple and Richmond, 1979; Carde and Laval, 1981; Begg *et al.*, 1987; Korbelik *et al.*, 1989; Skov and MacPhail, 1991). Substantial toxicity, especially nephro- and gastrointestinal toxicity, is associated with cisplatin. Carboplatin lacks most of the toxicities of cisplatin, especially when given as a prolonged infusion (Smit *et al.*, 1991), while it also shows radiosensitising effects in tumour cell lines (Douple *et al.*, 1985).

Preinduction of tolerance mechanisms by radiation have been associated with cisplatin cross-resistance (Hill *et al.*, 1990). Tolerance towards cisplatin can be induced by exposure to a low dose of this drug, leading to a platinum-resistant human small-cell cancer cell line, $GLC_4$-CDDP (Hospers *et al.*, 1988). It is not clear at the moment whether the radiosensitising effect of cisplatin and carboplatin is dependent on the inherent cellular sensitivity for these drugs. We studied in $GLC_4$-CDDP cross-resistance to radiation and compared possible radiosensitising properties of a combined treatment of platinum drugs and radiation in the resistant cell line with the sensitive parent cell line. Special attention is given to low- and moderate-dose radiation (0-8 Gy of X-rays) and drug treatments at clinically achievable concentrations, because treatment effects on the initial slope of the survival curve seem to be a significant factor in the clinical response of tumours to radiotherapy (Fertil and Malaise, 1981; Steel, 1993). The effects on cellular survival have quantitatively been expressed as ERs. Qualitative evaluations of the effects have been performed with the use of isobolograms according to the concept of Steel and Peckham (1979).

The outcome of the treatment was not only assayed in terms of cellular survival, but also as initial damage to DNA. Cisplatin does not itself produce double-strand breaks (dsbs), but it changes DNA conformationally (Chow *et al.*, 1994; Malinge *et al.*, 1994). Changes in DNA tertiary structure are linked to increased radiation sensitivity (Vaughan *et al.*, 1991), which has been associated with increased dsb frequencies, as measured with neutral filter elution analysis (Schwartz *et al.*, 1991). We used clamped homogeneous electric field (CHEF) gel electrophoresis, a sensitive assay for detecting dsbs in the low- and moderate-radiation dose range, to study the contribution of initial DNA damage to radiosensitisation.

Correspondence: HJM Groen, Department of Pulmonary Diseases, University Hospital Groningen, Oostersingel 59, 9713 EZ Groningen, The Netherlands
Received 24 April 1995; revised 27 July 1995; accepted 2 August 1995

### Materials and methods

### *Chemicals*

Cisplatin and carboplatin were obtained from Bristol-Myers Squibb, Weesp, The Netherlands. Roswell Park Memorial Institute (RPMI)-1640 medium was purchased from Gibco, Paisley, UK, and fetal calf serum (FCS) from Sanbio, Uden, The Netherlands. Dulbecco's modified Eagle (DME) and Ham's F12 media were obtained from Flow Laboratories, Irvine, UK, 3-(4,5-dimethylthiazol-2-yl)-2,5-diphenyltetrazolium bromide (MTT) and RNAse from Sigma, St Louis, MO, USA. Dimethyl sulphoxide was purchased from Merck, Darmstadt, Germany, and ethidium bromide from Serva, Heidelberg, Germany. Low-melt agarose MP was obtained from Boehringer Mannheim, Germany. Phosphate-buffered saline (PBS) consisted of 0.14 M sodium chloride, 2.7 mM potassium chloride, 6.4 mM disodium hydrogen phosphate dihydrate and 1.5 mM potassium hydrogen phosphate at pH 7.4.

### *Cell lines*

$GLC_4$, a human small-cell lung carcinoma cell line, and its 8.6-fold cisplatin-resistant subline $GLC_4$-CDDP were cultured in RPMI-1640 medium with 10% heat-inactivated FCS in a humidified atmosphere with 5% carbon dioxide at 37°C. To maintain a stable resistance level $GLC_4$-CDDP was cultured under constant challenge of a monthly dose of $75\ \mu g\ ml^{-1}$ cisplatin. The doubling times (mean ± s.e.) for $GLC_4$ and $GLC_4$-CDDP were 24 ± 1.4 and 25 ± 2.1 h respectively (Hospers *et al.*, 1988). Before starting experiments cells were cultured 3–6 weeks in a drug-free medium.

### *Drug and radiation exposure*

Cells in the exponential phase of growth were plated in $25\ cm^2$ flasks and incubated with cisplatin or carboplatin for 30 min, 4 or 24 h. Only cell culture with a viability of more than 90%, assessed by trypan blue exclusion, were used in experiments to limit apoptotic DNA fragmentation. Thereafter, cells were transferred to 96-well microtitre plates (100 µl of cell suspension per well) for MTT assay. In the combination experiments both platinum drugs were incubated for 30 min, 4 or 24 h before irradiation (0–8 Gy). Cells were transferred to tubes in a volume of 0.5 ml and either irradiated at room temperature before use in the MTT assay or irradiated at 0°C before use in CHEF electrophoresis. Radiation was carried out with Philips–Mueller MG X-ray source (200 kV, 15 mA, half valve layer 1.05 mm copper) at a dose rate of $5\ Gy\ min^{-1}$. Dosimetry was carried out using a $1\ cm^3$ Philips ionisation chamber (type 37489).

### *Microculture tetrazolium assay*

The assay is dependent on the cellular reduction of MTT by mitochondrial dehydrogenases of viable cells to a blue formazan product, which can be measured spectrophotometrically as described previously (Carmichael *et al.*, 1987*a*; De Vries *et al.*, 1989). Before the assay was performed the linear relationship of cell number to MTT formazan crystal formation was checked and cell growth studies were performed. Cells were in the exponential phase of growth at the moment of testing and at least two or three cell divisions should have taken place. Care was taken to select 1 day (at day 4) to test the cell survival for both cell lines under these conditions. Because the MTT assay is critically dependent on the number of cells plated, the amount of kill and the time of assay, these variables were checked at regular intervals. This was possible when we started at 3750 cells per well for $GLC_4$ and 15000 cells for $GLC_4$-CDDP for 4 days in 96-well microtitre plates (Nunc, Gibco, Paisley, UK). The differences in cell numbers were due to the fact that the $GLC_4$ cell line had a different lag time after treatment with drugs or radiation and because this cell line was metabolically more active. On the 4th day 20 µl of MTT solution (5 mg MTT $ml^{-1}$ phosphate-buffered saline; PBS) was added to each well for 3.75 h. After centrifugation of the plates (30 min, 180 *g*) supernatant was aspirated and dimethyl sulphoxide (100%, 200 µl) was pipetted to each well to dissolve crystals. The plate was immediately read at 520 nm using a scanning microtitre well spectrophotometer (Titertek Multiscan, Flow Laboratories). Controls consisted of media without cells (background extinction). At least three separate experiments were performed in quadruplicate at each tested platinum concentration and/or radiation exposure. The MTT assay can be used for measurement of survival effects after ionising radiation under specific laboratory conditions (Carmichael *et al.*, 1987*b*; Price and McMillan, 1990). Cell survival was defined as the growth of the treated cell populations compared with untreated control populations. Treatment consisted of radiation, platinum drugs or preincubation of either drug followed by radiation.

**Figure 1** Effect of carboplatin (**a**), cisplatin (**b**) and radiation (**c**) on the survival (mean ± s.e.m.) of $GLC_4$ and $GLC_4$-CDDP cell lines from all control experiments as measured by MTA. The 75% survival fraction is significantly different for both cell lines: (**a**) $P<0.001$; (**b**) $P<0.001$; (**c**) $P<0.05$). For each survival point three independent experiments in quadruplicate were performed. ■, $GLC_4$-CDDP; ▲, $GLC_4$.

### *CHEF electrophoresis*

A modified CHEF electrophoresis method as described earlier by Blöcher *et al.* (1989) was used. The percentage of electrophoretically extracted DNA was quantified by fluorescence-based image analysis of ethidium bromide-stained gels. Cells in exponential growth were concentrated to $2 \times 10^7\ ml^{-1}$ medium (DME/Ham's F12/FCS in 2:1:1) at 37°C and added to 1% low-melt agarose in PBS at 37°C (1:1) to form plugs. These plugs, containing $4.10^5$ cells, were irradiated in 200 µl of the same medium in Eppendorf vials at 0°C. Thereafter, the plugs were transferred to 96-well microtitre plates containing lysis buffer (proteinase K

0.5 mg ml$^{-1}$ in 0.5 M EDTA and 2% *N*-lauroylsarcosine at pH 7.6) initially for 2 h at 4°C and subsequently for 18 h at 37°C. After the plugs were rinsed with PBS, treatment with 0.2 mg ml$^{-1}$ RNAse in PBS followed for 50 min at 37°C. The plugs were washed twice with TBE buffer (45 mM Tris base, 45 mM boric acid and 2 mM EDTA at pH 8.3), loaded into the 0.5% agarose gel and sealed with low-melt agarose. CHEF gel electrophoresis (CHEF-DR II, Bio-Rad Laboratories, Richmond, USA) was carried out for 25 h at 14°C with a pulse time of 75 min at 40 V. The gel was stained with ethidium bromide (0.6 μg ml$^{-1}$ in TBE) for 12 h at 4°C and destained with TBE at least 2 h before analysing the gel. The plugs were removed carefully from the well and placed horizontally on top of the gel to avoid scattering of the bright fluorescence signal from the plug over the fragmented DNA. The reduced fluorescence signal from the plug was corrected for in each separate experiment by dividing the fluorescence signal from the plug in the well by the fluorescence signal of the plug lying horizontally on the gel. This correction factor was calculated from at least five extra plugs in the same gel as the original experiment was performed. Thereafter, this factor was used to correct all densitometric measurements coming from that gel. A linear relationship between the induction of dsb and DNA fragmentation has been observed before (Blöcher, 1990). The percentage of fragmented DNA expressed as DNA migrated out of the plug into the gel relative to the amount which remained into the plug was quantified using a densitometric analysis software package (Galai, Israel) on a CUE II image analysis system (Olympus) attached to a SIT camera. Details of this detection method were described previously (Rosemann *et al.*, 1993).

### *Data analysis*

Survival curves were established as base lines for both cell lines treated with cisplatin and carboplatin as well as after radiation exposure. Then survival curves for combination treatment were performed and adjusted for drug toxicity by starting each curve at a surviving fraction of 100%. From these curves enhancement ratios (ERs) were obtained by dividing the radiation dose without drug by the radiation dose in the presence of the drug, after correction for the drug-induced cytotoxicity, leading to a survival of 75%. This survival level has been arbitrarily selected to be within the initial slope of the survival curve and has been found to correlate with clinical outcome (Fertil and Malaise, 1981; Steel, 1993). All radiation and drug combinations were analysed with isobologram analysis. Additivity envelopes were calculated from the separate survival curves for radiation by X-rays and cisplatin or carboplatin. First, we considered a given amount of cell kill by cisplatin or carboplatin and then we measured the additional radiation dose needed to reach a survival level of 75%. This was performed by reading it off the radiation survival curve starting either from 75% survival (independent killing) or from the survival level already reached after cisplatin or carboplatin treatment (assuming dependent killing). Both describe an additivity envelope in relation to which the actual combined treatment data are compared (Steel and Peckham, 1979; Berenbaum, 1981).

Differences in cell survival and DNA fragmentation at different drug concentrations and irradiations were compared with Student's *t*-test for unpaired observations. Pearson's correlation was performed between ER and DNA dsbs occurring at the same drug inhibiting concentration and for the same incubation time. Only *P*-values $<0.05$ were considered significant.

### Results

### *Survival curves after drug or radiation treatment*

Survival curves for $GLC_4$ and $GLC_4$-CDDP cell lines were made for both cisplatin and carboplatin as well as for radiation in clincally relevant dose ranges (Figure 1a, b and c). In the $GLC_4$ and $GLC_4$-CDDP cell lines incubated with carboplatin the drug concentrations resulting in 25% growth inhibition ($IC_{25}$) were respectively 12.9 and 38.8 μM; the $IC_{50}$ values were 22.7 and 189 μM respectively (Figure 1a). The $IC_{25}$

**Figure 2** Effect of combined treatment of 24 h preincubation with carboplatin or cisplatin (both at $IC_{25}$) and radiation ($IC_{25}$) on both human lung cancer cell lines. (**a**) $GLC_4$-CDDP. ■, Untreated control; ▲, carboplatin and radiation. (**b**) $GLC_4$. ■, Untreated control; ▲, carboplatin and radiation. (**c**) $GLC_4$-CDDP. ■, Untreated control; ▲, cisplatin and radiation. (**d**) $GLC_4$. ■, Untreated control; ▲, cisplatin and radiation. $^*P<0.01$.

**Table I** Enhancement ratios for $GLC_4$ and $GLC_4$-CDDP lung cancer cell lines measured at $IC_{10}$, $IC_{25}$ and $IC_{50}$ concentrations of cisplatin (CDDP) and carboplatin (CBDCA) with radiation ($ID_{25}$).

| | | *Platinum incubation before irradiation* | | | | | |
|---|---|---|---|---|---|---|---|
| *Cell line* | *IC Drug* | *CDDP 30 min* | *CBDCA 30 min* | *CDDP 4 h* | *CBDCA 4 h* | *CDDP 24 h* | *CBDCA 24 h* |
| $GLC_4$ | $IC_{10}$ | 1.16(A) | 1.05(A) | 1.18(A) | 0.88(An) | 1.36(A) | 1.21(A) |
| $GLC_4$ | $IC_{25}$ | 1.21(A) | 1.04(A) | 1.26(A) | 0.93(A) | 1.39(A) | 1.11(A) |
| $GLC_4$ | $IC_{50}$ | 1.08(A) | 0.92(A) | 1.07(A) | 0.95(A) | 1.23(A) | 1.03(A) |
| $GLC_4$-CDDP | $IC_{10}$ | 1.33(A) | 0.67(A) | 0.99(A) | 1.13(A) | 1.09(A) | 1.11(A) |
| $GLC_4$-CDDP | $IC_{25}$ | 0.91(An) | 0.79(An) | 1.13(A) | 0.84(An) | 1.33(A) | 1.38(A) |
| $GLC_4$-CDDP | $IC_{50}$ | 0.89(An) | 0.70(An) | 0.99(A) | 0.89(An) | 0.99(A) | 0.96(An) |

Incubation time with each platinum drug was 30 min, 4 and 24 h before irradiation. Between brackets the isobolographic qualification of the interaction is described: A, addition; An, antagonism.

values of cisplatin in $GLC_4$ and $GLC_4$-CDDP cell lines were respectively 0.81 and 6.69 $\mu$M; the $IC_{50}$ values were 1.2 and 10.3 $\mu$M respectively (Figure 1b). With irradiation of $GLC_4$ and $GLC_4$-CDDP cell lines the radiation dose resulting in 25% growth inhibition and cell death ($ID_{25}$) were 0.87 and 2.25 Gy respectively; the $ID_{50}$ values were respectively 3.61 and 5.13 Gy. $GLC_4$-CDDP cell line showed cross-resistance to radiation (Figure 1c).

### *Effect of combined treatments*

Effects of cisplatin and carboplatin combined with irradiation in $GLC_4$ and $GLC_4$-CDDP were adjusted for drug toxicity (Figure 2a–d). Cisplatin ($IC_{25}$) combined with irradiation in the $GLC_4$ cell line significantly enhanced the radiation cytotoxicity ($P<0.01$); in $GLC_4$-CDDP the effect was not significant. The effect of equitoxic carboplatin ($IC_{25}$) with irradiation was also significant in $GLC_4$. In both cell lines the survival effect of carboplatin (30 min and 4 h preincubation) was less pronounced than with cisplatin. Higher cisplatin or carboplatin doses seemed to eliminate the enhanced cell toxicity.

Combination treatments showed a range of ERs at different concentrations and at different incubation times for both cisplatin and carboplatin (Table I). Cisplatin showed higher ERs than carboplatin. However, longer incubation with carboplatin showed an ER that approached cisplatin's effect in $GLC_4$-CDDP.

Whether these enhancing effects were qualitatively different with different doses and incubation times were evaluated by isobologram analysis. For all the combinations of cisplatin or carboplatin at three different incubation times we found mainly additive effects. In $GLC_4$ carboplatin ($IC_{10}$, preincubation 4 h) showed antagonism. In both cell lines at $IC_{50}$ levels of cisplatin (preincubation 24 h) antagonism was observed (ER in $GLC_4$ was 0.89 and in $GLC_4$-CDDP 0.71). No consistent synergism was observed in both cell lines, although in the $GLC_4$-CDDP cell line cisplatin approached synergism and low-dose carboplatin might be synergistic (Figure 3a and b). In particular the combination of radiation with cisplatin in $GLC_4$ was only additive.

### *Effects of the combined treatment on DNA fragmentation*

X-irradiation results in a linear increase of DNA extracted from the platinum-sensitive $GLC_4$ ($y = 2.18x + 14.32$, $r = 0.99$, $P<0.0001$) (Figure 4a). Non-irradiated cells could be extracted up to about 14% of total DNA. When radiation was given after a 24 h pretreatment with cisplatin ($IC_{50}$) or equitoxic dose of carboplatin, the linear regression lines representing extracted DNA in the dose range from 0–8 Gy were parallel to the line of irradiation alone. Treatment with cisplatin resulted in less extractable DNA at 0 Gy of X-rays. Almost the same picture emerged from experiments with the platinum-resistant cell line $GLC_4$-CDDP ($y = 2.83x + 10.16$,

**Figure 3** Isobolograms at 75% survival level for combinations of radiation and carboplatin (**a**) or cisplatin (**b**) both in the $GLC_4$-CDDP cell line. Both isobolograms show at least additive effects of the treatment components: in the middle portion of curve in (**a**) the radiation–carboplatin combination shows synergy and for curve (**b**) the radiation–cisplatin combination approaches synergy. □, Independent cell kill; △, dependent cell kill; ▲, combination of radiation and drug.

**Figure 4** (**a**) Modulation of low-dose radiation-induced DNA fragmentation (mean ± s.e.m.) by preincubating carboplatin or cisplatin in $GLC_4$ as measured by CHEF electrophoresis. DNA fragmentation from base line $GLC_4$ and $GLC_4$-CDDP in (**a**) and (**b**) did not differ significantly from each other ($P>0.05$). The decrease in fragmentation with carboplatin was not significant (except at 4 Gy in (**a**) $P<0.05$) and with cisplatin it was significantly decreased at all measurement points ($P<0.005$, at 0 and 1 Gy $P<0.05$). ■, Radiation only: ▲, radiation with cisplatin (24 h, $IC_{50}$); ▼, radiation with carboplatin (24 h, $IC_{50}$). (**b**) Modulation of low-dose radiation-induced DNA fragmentation (mean ± s.e.m.) by preincubating carboplatin or cisplatin in $GLC_4$-CDDP cell line as measured by CHEF electrophoresis. DNA fragmentation from base line $GLC_4$-CDDP did not differ from combined radiation with carboplatin ($P>0.05$). The decrease in fragmentation with carboplatin was not significant and with cisplatin it was significantly decreased at all measurement points ($P<0.005$, at 0 and 1 Gy $P<0.05$). ■, Radiation only; ▲, radiattion with cisplatin (24 h, $IC_{50}$); ▼, radiation with carboplatin (24 h, $IC_{50}$).

$r = 0.98$, $P < 0.003$) (Figure 4b). These cells showed less extractable DNA in the non-irradiated condition than observed in the $GLC_4$ cell line. The decrease in $GLC_4$-CDDP DNA fragmentation after preincubation with cisplatin was also more pronounced than in the $GLC_4$ cell line ($y = 2.30x + 10.50$, $r = 0.99$, $P < 0.001$ *vs* $y = 1.95x + 13.32$, $r = 0.98$, $P = 0.004$ in $GLC_4$), indicating that more crosslinks of DNA occurred in $GLC_4$-CDDP compared with the $GLC_4$ cell line at equitoxic dose.

A correlation between ERs and DNA dsbs occurring at the same drug dose ($IC_{50}$) and the same incubation time was not found.

### Discussion

Clinical trials with cisplatin and radiation in lung cancer showed better tumour control without enhanced normal tissue toxicity (Schaake-Koning *et al*., 1992). Addition of cisplatin to radiation in late-responding tissues such as a lung, kidney and rectum showed cytotoxic effects mainly explained by additive mechanisms. In early-responding tissues such as haematopoietic tissue, intestine and skin the kind of interaction is uncertain (Dewit, 1987). A substantial preferential radiosensitisation of hypoxic cells *in vitro* at low radiation doses (1–4 Gy) has been reported but not confirmed with low-dose multifraction radiation in oxic and hypoxic RIF-1 tumours (Skov and MacPhail, 1991; Sun and Brown, 1993). We studied combined treatment in human lung cancer cell lines in regard to the therapeutic index in lung cancer.

Treatment effects on the initial slope of the survival curve seemed to be a significant factor related to the clinical response of tumours to radiotherapy (Fertil and Malaise, 1981; Steel, 1993). This was the reason for selecting the arbitrary 75% survival level which was in the shoulder region of the survival curve, where the radiosensitising effects of both treatments were studied. We did not use survival values from the shoulder area of the survival curve extrapolated from the steep linear portion of the curve. The problem is the non-linear relationship between cell survival and radiation dose with broad limits of uncertainty in that range of the survival curve. Therefore, apart from the enhancement ratios, the more rigid isobolographic analysis was used to be sure to find a relevant effect.

As can be seen in Table I the ERs with 24 h platinum preincubation were compatible in both cell lines. The maximum ER approaches 1.4. This indicates that the radiosensitising capacity of the tumour cells seems not to be dependent on their sensitivity to platinum. Where we evaluated the enhancing effects with isobologram analysis, it showed that all enhancing effects are at least additive. No consistent synergy has been noticed, although it approaches synergy with prolonged platinum exposure. High platinum concentrations in the platinum-resistant $GLC_4$-CDDP cell line seems to abolish radiation toxicity. The mechanism behind this phenomenon is not yet clear. In other cell lines such as the platinum-sensitive RIF-1 and Chinese hamster cell line V79 enhanced radiation-induced cytotoxicity also suggests a role for cisplatin as well as for prolonged carboplatin exposure in combination with radiotherapy (Dewit, 1987; Skov and MacPhail, 1991). Many animal studies, but not all, have shown more than additive effects with high radiation doses (Stephens *et al*., 1986; Coughlin and Richmond, 1989).

Cisplatin causes a range of DNA lesions of which DNA intrastrand links are considered the most biologically relevant for the process of cell kill. Radiation-induced cell kill is related to DNA double-strand breaks (Frankenberg-Schwager, 1990). Several ways of interaction between both treatments may occur on the DNA level. Differences in residual DNA breaks may be the result of inhibition DNA repair as a result of cisplatin-induced DNA crosslinks. Alternatively, reduced residual double-strand breaks may be observed owing to the priming of DNA repair processes by platinum pretreatment, explaining differences in cell kill between platinum-sensitive and -resistant cell lines. At the moment research is focused on the DNA repair capacity (rates of repair and residual damage) of tumour cells pretreated with platinum compounds. The third possibility is DNA or chromatin conformational changes by different agents, associated with increased radiation sensitivity, explaining the interaction between both treatments. An increased DNA accessibility for DNAase I, or an enhanced ability to adopt positive DNA supercoiling has been observed in a radiation-sensitive cell line as compared with a radiation-resistant cell line (Milner *et al*., 1993). Also, DNA unfolding by removal of magnesium or histon proteins (Heussen *et al*., 1987), or in actively transcribed DNA sustains more DNA double-strand breaks than does bulk DNA, possibly as a result of better access to locally produced radicals (Chiu *et al*., 1986). Platinum may also change DNA conformation (Chow *et al*., 1994; Malinge *et al*., 1994) and therefore may influence induced DNA double-strand formation, although cisplatin itself does not produce double-strand breaks. In this study enhanced radiosensitivity by platinum exposure was probably not caused by an enhancement of radiation-induced dsbs in DNA.

Because longer incubations with both platinum drugs led to ERs approaching synergism it was of interest to explore the possibility of an enhanced formation of dsbs in DNA directly after radiation. However, such an enhanced formation was not found. In most cases platinum pretreatment led to a decreased DNA extractability probably caused by extensive cross-linking. In this study radiation cross-resistance in the platinum resistant cell line was observed. The ability of this subline to form more crosslinks with platinum as compared with the sensitive cell line at equitoxic dose is probably due to the higher platinum dose used in the resistant cell line. Whether DNA accessibility for cisplatin has changed is unknown.

In conclusion, the platinum-resistant lung cancer cell line is cross-resistant to radiation and shows less DNA fragmentation after cisplatin exposure than the sensitive cell line. The radiosensitising capacity of these lung cancer cell lines is neither dependent on their platinum sensitivity nor on initial DNA damage by radiation. Longer platinum exposure seems to increase ERs, but they only approach synergism. Further exploration of prolonged exposure to carboplatin covering the whole radiation period is warranted in lung cancer patients.

### References

BEGG AC, VAN DER KOLK PJ, EMONDT J AND BARTELINK H. (1987). Radiosensitization in vitro by *cis*-diammine (1,1-cyclobutanecarboxylato) platinum(II) (carboplatin, JM8) and ethylenediammine-malonatoplatinum(II) (JM40). *Radiother. Oncol.*, **9**, 157–165.

BERENBAUM MC. (1981). Criteria for analyzing interactions between biologically active agents. *Adv. Cancer Res.*, **35**, 269–290.

BLÖCHER D. (1990). In CHEF electrophoresis a linear induction of dsb corresponds to a non-linear fraction of extracted DNA with dose. *Int. J. Radiat. Biol.*, **57**, 7–12.

BLÖCHER D. EINSPENNER M AND ZAJACKOWSKI J. (1989). CHEF electrophoresis, a sensitive technique for the determination of DNA double-strand breaks. *Int. J. Radiat. Biol.*, **56**, 437–448.

CARDE P AND LAVAL F. (1981). Effects of cis-dichlorodiammine platinum II and X-rays on mammalian cell survival. *Int. J. Radiat. Oncol. Biol. Phys.*, **7**, 929–933.

CARMICHAEL J, DEGRAFF WG, GAZDAR AF, MINNA JD AND MITCHELL JB. (1987*a*). Evaluation of a tetrazolium-based semiautomated colorimetric assay: assessment of chemosensitivity testing. *Cancer Res.*, **47**, 936–942.

CARMICHAEL J, DEGRAFF WG, GAZDAR AF, MINNA JD AND MITCHELL JB. (1987*b*). Evaluation of a tetrazolium-based semiautomated colorimetric assay: assessment of radiosensitivity. *Cancer Res.*, **47**, 943–946.

CHIU SM, FRIEDMAN LR, SOKANY NM, XUE LY AND OLEINICK NL. (1986). Nuclear matrix proteins are crosslinked to transcriptionally active gene sequences by ionizing radiation. *Radiat. Res.*, **107,** 24–38.

CHOW CS, WHITEHEAD JP AND LIPPARD SJ. (1994). HMG domain proteins induce sharp bends in cisplatin-modified DNA. *Biochemistry*, **33,** 15124–15130.

COUGHLIN CT AND RICHMOND RC. (1989). Biologic and clinical developments of platinum combined with radiation: concepts, utility, projections for new trials, and the emergence of carboplatin. *Semin. Oncol.*, **16,** 31–43.

DE VRIES, EGE, MEIJER C, TIMMER-BOSSCHA H, BERENDSEN HH, DE LEIJ L, SCHEPER RJ AND MULDER NH. (1989). Resistance in three human small cell lung cancer cell lines established from one patient during clinical follow-up. *Cancer Res.*, **49,** 4175–4178.

DEWIT L. (1987). Combined treatments of radiation and cisdiamminechloroplatinum (II): a review of experimental and clinical data. *Int. J. Radiat. Oncol. Biol. Phys.*, **13,** 403–426.

DOSORETZ DE, KATIN MJ, BLITZER PH, RUBENSTEIN JH, SALENIUS S, RASHID M, DOSANI RA, MESTAS G, SIEGEL AD, CHADHA TT, CHANDRAHASA T, HANNAN SE, BHAT SB AND METKE MP. (1992). Radiation therapy in the management of medically inoperable carcinoma of the lung: results and implications for future treatment strategies. *Int. J. Radiat. Oncol. Biol. Phys.*, **24,** 3–9.

DOUPLE EB AND RICHMOND RC. (1979). Radiosensitization of hypoxic tumour cells by cis- and trans-dichlorodiammineplatinum (II). *Int. J. Radiat. Oncol. Biol. Phys.*, **5,** 1369–1372.

DOUPLE EB, RICHMOND RC, O'HARA JA AND COUGHLIN CT. (1985). Carboplatin as a potentiator of radiation therapy. *Cancer Treat. Rev.*, **12,** 111–124.

FERTIL B AND MALAISE EP. (1981). Inherent radiosensitivity as a basic concept for human tumor radiotherapy. *Int. J. Radiat. Oncol. Biol. Phys.*, **7,** 621–629.

FRANKENBERG-SCHWAGER M. (1990). Induction, repair and biological relevance of radiation-induced DNA lesions in eukaryotic cells. *Radiat. Environ. Biophys.*, **29,** 273–292.

HEUSSEN C, NACKERDIEN Z, SMIT BJ AND BOHM L. (1987). Irradiation damage in chromatin isolated from V-79 Chinese hamster lung fibroblasts. *Radiat. Res.*, **110,** 84–94.

HILL BT, SHELLARD SA, HOSKING LK, FICHTINGER-SCHEPMAN AMJ AND BEDFORD P. (1990). Enhanced repair and tolerance of DNA damage associated with resistance to cis-diammine-dichloroplatinum (II) after in vitro exposure of a human teratoma cell line to fractionated X-irradiation. *Int. J. Radiat. Oncol. Phys.*, **19,** 75–83.

HOSPERS GAP, MULDER NH, DE JONG B, DE LEIJ L, UGES DRA, FICHTINGER-SCHEPMAN AMJ AND DE VRIES EGE. (1988). Characterization of a human small cell lung carcinoma cell line with acquired cisplatin resistance in vitro. *Cancer Res.*, **48,** 6803–6807.

KORBELIK M AND SKOV K. (1989). Inactivation of hypoxic cells by cisplatin and radiation at clinically relevant doses. *Radiat. Res.*, **119,** 145–156.

MALLINGE JM, PEREZ C AND LENG M. (1994). Base sequence-independent distorsions induced by interstrand cross-links in cis-diamminedichloroplatinum (II)-modified DNA. *Nucleic Acids Res.*, **22,** 3834–3839.

MILNER AE, GORDON DJ, TURNER BM AND VAUGHAN ATM. (1993). A correlation between DNA-nuclear matrix binding and relative radiosensitivity in two human squamous cell carcinoma cell lines. *Int. J. Radiat. Biol.*, **63,** 13–20.

PRICE P AND MCMILLAN TJ. (1990). Use of the tetrazolium assay in measuring the response of human tumor cells to ionizing radiation. *Cancer Res.*, **50,** 1392–1396.

ROSEMANN M, KANON B, KONINGS AWT AND KAMPINGA HH. (1993). An image analysis technique for detection of radiation-induced DNA fragmentation after CHEF electrophoresis. *Int. J. Radiat. Biol.*, **64,** 245–249.

SCHAAKE-KONING C, VAN DEN BOGAERT W, DALESIO O, FESTEN J, HOOGENHOUT J, VAN HOUTTE P, KIRKPATRICK A, KOOLEN M, MAAT B, NIJS A, RENAUD A, RODRIGUS P, SCHUSTER-UITTERHOEVE L, SCULIER JP, VAN ZANDWIJK N AND BARTELINK H. (1992). Effects of concomitant cisplatin and radiotherapy on inoperable non-small-cell lung cancer. *N. Engl. J. Med.*, **326,** 524–530.

SCHWARTZ JL, MUSTAFI R, BECKETT MA, CZYZEWSKI EA, FARHANGI E, GRDINA DJ, ROTMENSCH J AND WEICHSELBAUM RR. (1991). Radiation-induced DNA double-strand break frequencies in human squamous cell carcinoma cell lines of different radiation sensitivities. *Int. J. Radiat. Biol.*, **59,** 1341–1352.

SKOV K AND MACPHAIL S. (1991). Interaction of platinum drugs with clinically relevant X-ray doses in mammalian cells: a comparison of cisplatin, carboplatin, iproplatin, and tetraplatin. *Int. J. Radiat. Biol. Phys.*, **20,** 221–225.

SMIT EF, WILLEMSE PHB, SLEIJFER D.TH, UGES DRA, POSTMUS PE, MEIJER S, TERHEGGEN PMAB, MULDER NH AND DE VRIES EGE. (1991). Continuous infusion carboplatin on a 21-day schedule: a phase I and pharmacokinetic study. *J. Clin. Oncol.*, **9,** 100–110.

STEEL GG. (1993). The radiobiology of tumours. In Steel GG (ed.) pp. 116–119. *Basic Clinical Radiobiology*. Edward Arnold: London.

STEEL GG AND PECKHAM MJ. (1979). Exploitable mechanisms in combined radiotherapy: the concept of additivity. *Int. J. Radiat. Oncol. Biol. Phys.*, **5,** 85–91.

STEPHENS TC, ADAMS K, PEACOCK JH AND STEEL GG. (1986). Temporal interactions in the Lewis lung tumour between cytotoxic drugs and acute or fractionated radiotherapy. *Radiother. Oncol.*, **5,** 137–146.

SUN JR AND BROWN JM. (1993). Lack of differential radiosensitization of hypoxic cells in a mouse tumor at low radiation doses per fraction by cisplatin. *Radiat. Res.*, **133,** 252–256.

VAUGHAN ATM, MILNER AE, GORDON DG AND SCHWARTZ JL. (1991). Interaction between ionizing radiation and supercoiled DNA within human tumour cells. *Cancer Res.*, **51,** 3857–3861.